# Calcifying fibrous tumour of the pleura without gross calcification

**DOI:** 10.1002/rcr2.1365

**Published:** 2024-05-01

**Authors:** Hwan Jin Lee, Jae Seok Jeong, Yong Chul Lee

**Affiliations:** ^1^ Department of Internal Medicine, Research Center for Pulmonary Disorders Jeonbuk National University Medical School Jeonju Republic of Korea; ^2^ Research Institute of Clinical Medicine of Jeonbuk National University‐Biomedical Research Institute of Jeonbuk National University Hospital Jeonju Republic of Korea; ^3^ Laboratory of Respiratory Immunology and Infectious Diseases, Korea Zoonosis Research Institute Jeonbuk National University Iksan Republic of Korea; ^4^ Respiratory Drug Development Research Institute Jeonbuk National University Medical School Jeonju Republic of Korea

**Keywords:** atypical, calcifying fibrous tumour, calcifying fibrous tumour, calcifying fibrous tumour of the pleura

## Abstract

Calcifying fibrous tumours of the pleura (CFTP) typically appear as calcified, non‐enhancing lesions on chest CT scans. However, enhancing pleural lesions can mimic malignancy like mesothelioma. We report a rare case that enhancing pleural thickening, confirmed as CFTP through pathological examination, despite the absence of visible calcification on radiological imaging.

## Graphical abstract

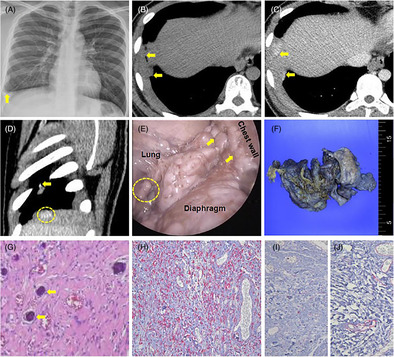

A 19‐year‐old male was referred to our pulmonology clinic for pleural thickening of right side on chest x‐ray, which was incidentally found on the Army's enlistment examination (Figure 1A, yellow arrow). He had smoked 1–2 cigarettes per day for 1 year. He had no symptoms and no exposure to asbestos. Non‐enhanced chest computed tomography (CT) showed no signs of calcification for the pleural thickening lesions in cross sectional view (Figure 1B, yellow arrows) Contrast‐enhanced chest CT showed diffuse thickening of right pleural space involving visceral pleura and right diaphragm with heterogeneous enhancement (Figure 1C, yellow arrows and Figure 1D, yellow arrow and yellow circle). Video‐assisted thoracoscopic surgery (VATs) revealed multiple soft mass lesions with irregular shapes at the entire right pleural space extending to diaphragmatic area and visceral pleura (Figure 1E, yellow arrows and yellow circle). The specimen resected from the surgery measured 12.5 × 9.6 × 3.7 cm and showed irregular surface and thickened pleura about 1.4 cm thick (Figure 1F). Haemotoxylin and Eosin staining of the specimen disclosed psamommatous calcification (Figure 1G, yellow arrows). Immunohistochemical staining showed positivity of spindle‐shaped cells only for vimentin (Figure 1H) and negativity both for S‐100 and CD‐34 (Figure 1I,J, respectively) that are comparable with calcifying fibrous tumour. A follow‐up chest CT at 7 months later showed no local recurrence suggesting complete surgical resection.

**FIGURE 1:**
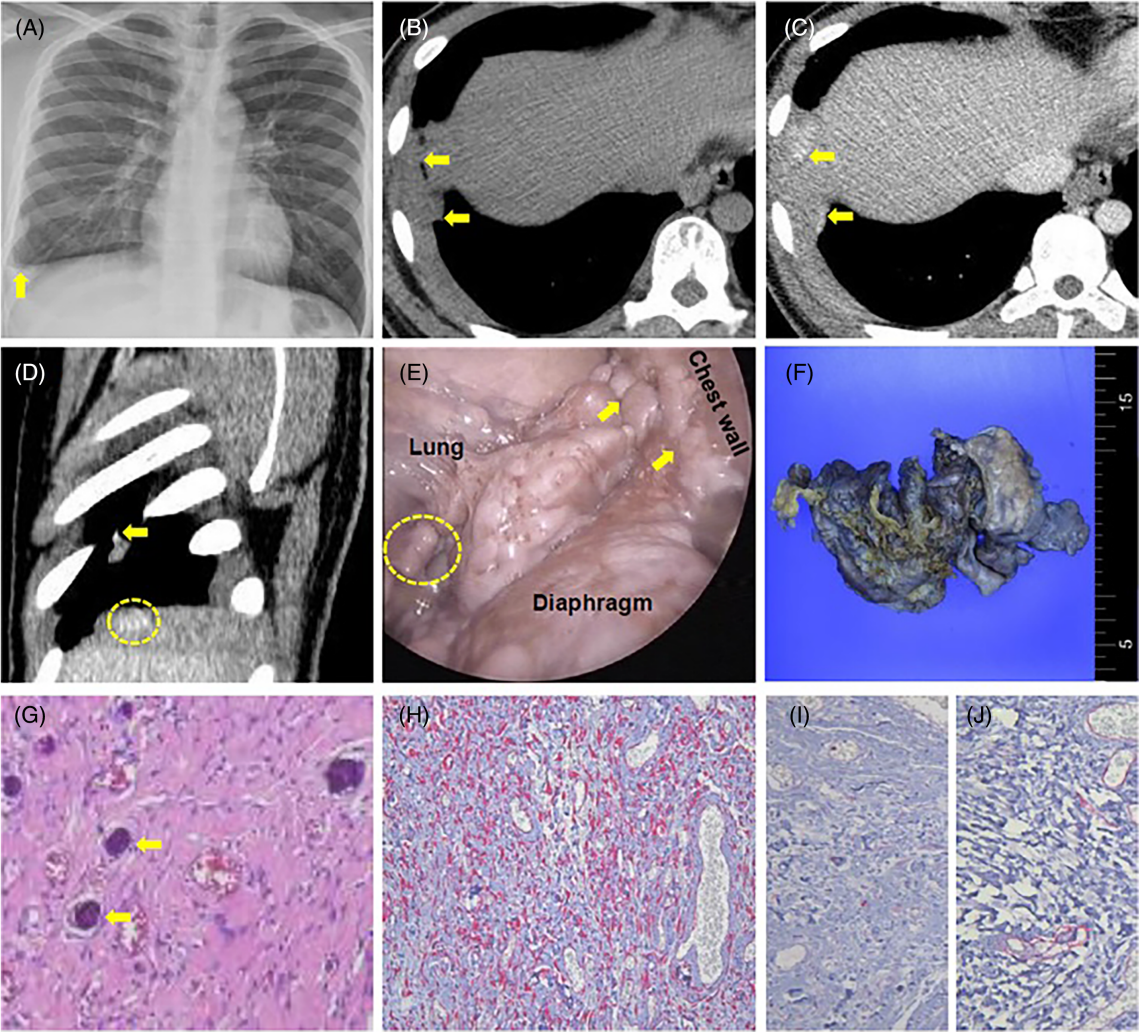
(A) Simple chest x‐ray shows right pleural thickening (arrow). (B) Cross‐sectional view of pre‐phase of contrast‐enhanced chest computed tomography (CT) shows right diffuse pleural thickening without lesions suggesting calcification (arrows). (C and D) Contrast‐enhancing lesions are shown on cross‐sectional view (C, arrows) and sagittal sectional view (D, arrow and dotted circle) of the chest CT. (E) Video‐assisted thoracoscopic surgery reveals multiple soft mass lesions with irregular shapes involving right visceral pleura, diaphragm and chest wall (arrows and dotted circle). (F) The specimen resected from the surgery measures 12.5 × 9.6 × 3.7 cm with irregular surface. (G) Psamommatous calcifications are seen on H&E staining (arrows). (H‐J) Immunohistochemical staining of the specimen is only positive for vimentin (H) and negative both for S‐100 (I) and CD‐34 (J).

To our best knowledge about calcifying fibrous tumour of the pleura (CFTP), calcification was demonstrated on radiologic image assessment in almost all reported cases.^1^, ^2^ Although chest CT slice thickness, 3 mm for this case, should be considered for interpreting chest CT results, interestingly, this case showed no apparent sign of calcification either on chest x‐ray or chest CT. Based on our experience, physicians need to suspect the possibility of CFTP with pleural thickening and features of malignancy irrespective of gross calcification on chest x‐ray and/or CT.

## AUTHOR CONTRIBUTIONS

Hwan Jin Lee was involved in writing original draft, writing–review and editing and final approval of the manuscript. Young Chul Lee and Jase Seok Jeong were involved in writing original draft, reviewing, and the final approval of the manuscript and served as a supervisors throughout the manuscript writing process.

## FUNDING INFORMATION

This research was supported by the Bio & Medical Technology Development Program of the National Research Foundation (NRF) funded by the Korean government (MSIT) (No. RS‐2023‐00236157; JSJ). This paper was supported by Fund of Biomedical Research Institute, Jeonbuk National University Hospital. No competing financial interests exist.

## CONFLICT OF INTEREST STATEMENT

None declared.

## ETHICS STATEMENT

The authors declare that appropriate written informed consent was obtained for the publication of this manuscript and accompanying images.

## Data Availability

The data that support the findings of this study are available from the corresponding author upon reasonable request.
